# Mr. Drew, on Inoculation

**Published:** 1805-12

**Authors:** Walter Drew

**Affiliations:** No. 70, Gower Street


### *To the Editors of the Medical and Physical Journal.*

Gentlemen,

As the two following cases exhibit rather a remarkable coincidence of failure, in the same family, of variolation as well as vaccination, on this account I take the liberty of relating to you the particulars, conceiving you may probably deem them worthy to be inserted in your Medical Journal.

In the year 1799, I inoculated with small-pox matter Joseph Chitty, th n about a year old; the arm went through through the regular process of maturation; symptomatic fever of a mild nature took place about the 9th day, and the usual evanescing course followed. The father of the child asserts that one pustule had formed on the loins; but on this circumstance I, at this period, cannot bring my recollection to bear; however, in appearance, the effect of this inoculation had been sufficiently complete, to satisfy me in giving it as my opinion, that there was ample security against the contagion of small-pox in future.

This child, about the beginning of the last month, was seized with small-pox, and narrowly escaped with his life.

The brother of this child, the only one besides in the family, I vaccinated last spring twelve months, then about six months old; the arm of this child also exhibited all those criteria which vaccination generally puts on, such as the hardened phlegmonic base and inflammatory areola, encompassing the pustule from the 9th to the 11th day, and its gradual change to the dark brown prominent scab, which adhered a long time, leaving behind an indelible impression in the arm, such as in appearance, a second time, to enable me to warrant safety from small-pox influence. Again have I been disappointed, for, about sixteen days from the time his brother became affected, this child was seized with eruptive fever to a very high degree, and small-pox of the distinct kind followed.

The former of these cases was attended by Mr. Simpson, of Harpur-Street, Red Lion Square; and both have been witnessed by Mr. Ring, Doctors Willan, Pearson, Walker, of the Central Jennerian Institution, and others. The latter named gentleman had been sanguine, like myself, from the appearance of impression after vaccination, in assuring the parents, they might entertain no fear respecting its power of resisting the contagion from their elder child; *unfortunately*, the fact turned out otherwise. I think it material to state, that I have inoculated three patients with great attention, with matter taken from the last mentioned case, and have not been able to produce small-pox.

I am, &c.

WALTER DREW.

No. 70, *Gower Street*,

*Oct.* 14, 1805.

